# STAT3‐mediated *MLST8* gene expression regulates cap‐dependent translation in cancer cells

**DOI:** 10.1002/1878-0261.12735

**Published:** 2020-06-29

**Authors:** Hyunji Lee, Hyunjung Chin, Hyeyoung Kim, Hosung Jung, Daekee Lee

**Affiliations:** ^1^ Department of Life Science Ewha Womans University Ewhayeodae-gil 52, Seodaemun-gu Seoul South Korea; ^2^ Department of Anatomy, and Brain Research Institute Yonsei University College of Medicine Seoul South Korea

**Keywords:** 4E‐BP1, cross‐talk, MLST8, mTORC1, STAT3

## Abstract

Signal transducer and activator of transcription 3 (STAT3) regulates cell growth, cell survival, angiogenesis, metastasis of cancer cells, and cancer immune evasion by regulating gene expression as a transcription factor. However, the effect of STAT3 on translation is almost unknown. We demonstrated that STAT3 acts as a trans‐acting factor for *MLST8* gene expression and the protein level of mLST8, a core component of mechanistic target of rapamycin complex 1 and 2 (mTORC1/2), positively regulates the mTORC1/2 downstream pathways. Suppression of STAT3 by siRNA attenuated 4E‐BP1 phosphorylation, cap‐dependent translation, and cell proliferation in a variety of cancer cells. In HCT116 cells, *STAT3* knockdown‐induced decreases in 4E‐BP1 and AKT phosphorylation levels were further attenuated by *MLST8* knockdown or recovered by mLST8 overexpression. *STAT3* knockdown‐induced G2/M phase arrest was partially restored by co‐knockdown of *4EBP1*, and the attenuation of cell proliferation was enhanced by the expression of an mTORC1‐mediated phosphorylation‐defective mutant of 4E‐BP1. ChIP and promoter mapping using a luciferase reporter assay showed that the −951 to −894 bp of *MLST8* promoter seems to include STAT3‐binding site. Overall, these results suggest that STAT3‐driven *MLST8* gene expression regulates cap‐dependent translation through 4E‐BP1 phosphorylation in cancer cells.

## Graphical abstract

Although signal transducer and activator of transcription 3 (STAT3) and mTOR pathways play crucial roles in tumorigenesis, studies are underway to investigate cross‐talk between the two pathways in cancer cells. We have demonstrated that STAT3 acts as a trans‐acting factor for *MLST8* gene expression and the protein level of mLST8, a core component of mechanistic target of rapamycin complex 1 and 2, positively regulates cap‐dependent translation through 4E‐BP1 phosphorylation in cancer cells.

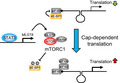

**Abbreviations**
4E‐BPs: eIF4E‐binding proteins
eIFs: eukaryotic initiation factors
IP: immunoprecipitation
m^7^GTP: 7‐methylguanosine
mTOR: mechanistic target of rapamycin
mTORC1: mechanistic target of rapamycin complex 1
mTORC1/2: mechanistic target of rapamycin complex 1 and 2
mTORC2: mechanistic target of rapamycin complex 2
qRT–PCR: quantitative reverse transcription and real‐time PCR
S6K: ribosomal protein S6 kinase
siRNA: small interfering RNA
STAT3: signal transducer and activator of transcription 3

## 1 Introduction

Signal transducer and activator of transcription 3 (STAT3), the most studied member of the STAT protein family, is a transcription factor which transmits signals from cytokines and growth factors, translocates to the nucleus as a phospho‐STAT3 dimer, and activates the expression of target genes (Darnell, 1997). STAT3 signaling is involved in the progression of the cell cycle and the prevention of apoptosis by upregulating the expression of cell growth and survival proteins (Huynh et al., 2017). STAT3 is constitutively active in a variety of human malignancies and regulates the expression of target genes involved in tumorigenesis and cancer progression (Cao et al., 2014; Johnson et al., 2018; Yu et al., 2014). Inhibition of STAT3 in wide range of cancer cell lines with small molecular inhibitors, dominant‐negative mutants, and small interfering RNA (siRNA) results in a decline in cell proliferation, indicating that STAT3 is a potential target for anticancer therapies (Lin et al., 2011; Lin et al., 2005; Ni et al., 2000; Zhang et al., 2008).

The activation of the PI3K‐AKT or MAPK pathways by nutrients and growth factors culminates in the regulation of the protein mechanistic target of rapamycin (mTOR) which coordinates the growth, survival, proliferation, and metabolism of cells (Blenis, 2017; Saxton and Sabatini, 2017). mTOR forms two distinct complexes, mTORC1 and mTORC2. mTORC1 contains the core components mLST8 and Raptor, and two inhibitory subunits DEPTOR and PRAS40, while mTORC2 contains the core components mLST8 and Rictor, an inhibitory subunit DEPTOR, and stimulatory subunits Protor1/2 and mSin1 (Saxton and Sabatini, 2017). Transcriptional activation by transcription factors, as well as general mRNA translation, is known to be increased in tumor cells (Blenis, 2017; Silvera et al., 2010; Sonenberg and Hinnebusch, 2009). Translation of mRNA is mainly exerted at translation initiation through the coordinated actions of members of the eukaryotic initiation factor (eIF) family. The cap‐binding protein eIF4E, together with helicase eIF4A and scaffold protein eIF4G, forms eIF4F complexes, which play an important role in the regulation of cap‐dependent translation. eIF4F is negatively regulated by eIF4E‐binding proteins (4E‐BPs), which interact with eIF4E to prevent eIF4G binding (Richter and Sonenberg, 2005). mTORC1 signaling directly governs the cell growth by regulating protein synthesis *via* the phosphorylation of 4E‐BPs and ribosomal protein S6 kinase (S6K), whereas mTORC2 signaling regulates cell survival, proliferation, and migration *via* the phosphorylation of AKT(S473) and PKC (Saxton and Sabatini, 2017).

Recent reviews have demonstrated that many cancers have increased mTOR activity due to deregulation of upstream and downstream mTOR signal pathways (Blenis, 2017; Saxton and Sabatini, 2017; Seeboeck et al., 2019). mTORC1/2 core components and regulators are also involved in tumorigenesis in a variety of cancers. Increased activation of mTORC1/2 pathways due to *MTOR* mutations has been reported in a range of cancers (Grabiner et al., 2014). mLST8, a core component of both mTORC1 and mTORC2, associates with the kinase domain of mTOR and may stabilize the active site (Xu et al., 2013). mLST8 is upregulated in human colon and prostate cancer cells, in which it contributes to tumor growth by regulating mTORC1/2 activity (Kakumoto et al., 2015). Raptor is overexpressed in prostatic adenocarcinomas (Evren et al., 2011), and knockdown of *RAPTOR* induces attenuation of mTORC1 kinase activity, followed by reduction in S6K and 4E‐BP1 phosphorylation and cell growth (Fuhler et al., 2009; Kim et al., 2002). Expression of DEPTOR, a negative regulator of mTORC1/2, is known to be low in many cancer cells (Peterson et al., 2009), however, DEPTOR acts as a tumor suppressor or oncogene, depending on the cellular context of cancers (Catena and Fanciulli, 2017). Hyperphosphorylation of PRAS40, resulting in dissociation from mTORC1 and enhanced mTOR activation, has been found in a variety of cancers, including melanoma, prostate cancer, stomach cancer, and non‐small‐cell lung cancer (Lv et al., 2017). Overexpression of Rictor promotes glioma cell growth and motility by elevation of mTORC2 activity (Masri et al., 2007). Downregulation of mSin1 inhibits hepatocellular carcinomas invasion and metastasis *via* mTORC2 inactivation (Xu et al., 2013). However, the role of Protor1/2 in cancers has not been studied in depth.

Although both the STAT3 and mTOR pathways play crucial roles in tumorigenesis, studies are underway to investigate cross‐talk between the two pathways in cancer cells. STAT3‐S727 phosphorylation by ciliary neurotrophic factor treatment in neuroblastoma cells has been reported to be induced by mTOR kinase (Yokogami et al., 2000), but the mechanism of mTOR pathway regulation by the STAT3 pathway is unknown. Here, we show that knockdown of *STAT3* by siRNA treatment induces cell cycle arrest and apoptosis by a decrease in cap‐dependent translation in human cancer cells. We also demonstrated that *STAT3* knockdown‐induced attenuation of cap‐dependent translation is mediated by a decline in mLST8 expression, followed by downregulation of 4E‐BP1 phosphorylation. Overall, we suggest that mLST8 constitutes a potential target for cancer therapy as a cross‐road between STAT3 and the mTOR pathway.

## 2 Materials and methods

### 2.1 Reagents

The mutant 4E‐BP1 expression vector (p4EBP1‐4A) was obtained from Addgene (#38240) (Thoreen et al., 2012). The *MLST8* cDNA expression vector (hMU000234) in which full‐length cDNA was inserted into a pCMV‐SPORT6 vector was obtained from KHGB (Daejeon, Korea). All chemicals were purchased from Sigma‐Aldrich (St. Louis, MO, USA), unless otherwise stated.

### 2.2 Cell culture

Human cancer cells (origin), A549 (lung), ACHN (kidney), HCT116 (colon), LNCaP (prostate), and MDA‐MB‐231 (breast), were obtained from ATCC (Manassas, VA, USA) and KCLB (Seoul, Korea). A549, LNCaP, and MDA‐MB‐231 cells were grown in RPMI1640 medium, HCT116 cells were grown in McCoy's 5A medium, and ACHN cells were grown in DMEM medium at 37 °C in a humidified atmosphere with 5% CO_2_. All media were supplemented with 10% FBS. Both floating and adherent cells were harvested for the cell proliferation assays and FACS analysis, whereas adherent cells were washed twice with cold PBS and immediately stored at −80 °C for RNA and protein extraction or luciferase assay.

### 2.3 Transfection of cells with siRNA or DNA

Cells were seeded in six‐well plates for 18 h before transfection with siRNA. Lipofectamine RNAiMAX transfection reagent was used for siRNA transfection according to the manufacturer's instructions (Thermo Fisher, Waltham, MA, USA). ON‐TARGETplus Non‐targeting Pool from Dharmacon Inc. (Lafayette, CO, USA) was used for control siRNA (siCTRL). For the negative control, an equal amount of siCTRL was added to adjust the concentration of total siRNA. Gene‐specific siRNA duplexes with additional 3′ UU overhangs were synthesized by Genolution Pharmaceuticals (Seoul, Korea). The siRNA target sequences are shown in Table S1. For DNA transfection, cells were transfected with 2.5 μg of pcDNA3.1 vector (Thermo Fisher) or cDNA expression vector or using LT1 reagent (Mirus Bio, Madison, WI, USA). Doxycycline (2 μg·mL^−1^) was treated into cells for 48 h.

### 2.4 Cell proliferation assay

For cell proliferation assay, 5 × 10^3^ (A549, ACHN, HCT116, and MDA‐MB‐231) or 10 × 10^3^ (LNCaP) cells were plated on 96‐well plates 18 h before treatment with 5 nm of siRNA. Cell proliferation was determined with WST‐1 colorimetric assay (Lee et al., 2009) or counting the number of live cells as described previously (Lee et al., 2014).

### 2.5 Cell cycle analysis with FACS

Cell cycle was analyzed using a flow cytometer, FACSCalibur, from BD Bioscience (San Jose, CA, USA) as described previously (Lee et al., 2014).

### 2.6 Preparation of cell extracts and western blot analysis

Cell extracts were prepared by scraping the cells with 60–80 μL of cold lysis buffer (Lee et al., 2014) and storing on ice for 10 min. Clearing of the homogenates, protein quantification, SDS/PAGE, western blotting, and quantification of blots were performed as described previously (Lee et al., 2009). The antibodies (Catalog number) against 4E‐BP1 (#9644), phospho‐4E‐BP1(T37/46) (#2855), phospho‐4E‐BP1(S65) (#9451), phospho‐4E‐BP1(T70) (#9455), 4E‐BP2 (#2845), AKT (#4691), phospho‐AKT(T308) (#2965), phospho‐AKT(S473) (#9271), eIF4A (#2013), eIF4A1 (#2490), eIF4B (#3592), phospho‐eIF4B(S422) (#3591), eIF4E (#2067), eIF4G (#2469), eIF4H (#3469), mLST8 (#3274), Raptor (#2280), Rictor (#2214), p70S6K (#2708), phospho‐p70S6K (T389) (#9205), STAT1 (#9172), phospho‐STAT1(Y701) (#9171), STAT3 (#4904), phospho‐STAT3(Y705) (#9131), phospho‐STAT3(S727) (#9134), mTOR (#2983), phospho‐mTOR(S2448) (#5536), and phospho‐mTOR(S2481) (#2974) were obtained from Cell Signal Technology (Danvers, MA, USA); GAPDH (#CSB‐MA000071M0m) was obtained from Cusabio (Houston, TX, USA).

### 2.7 Immunoprecipitation

HNTG buffer (20 mm HEPES, 150 mm NaCl, 0.1% Triton X‐100, 10% glycerol) was supplemented with protease inhibitors (1 mm PMSF, 10 μg·mL^−1^ of leupeptin, and 10 μg·mL^−1^ of aprotinin) and phosphatase inhibitors (1 mm Na_3_VO_4_, 1 mm NaF, and 10 mm β‐glycerophosphate) just before use. Cell extract (0.2–0.5 mg) in 0.4 mL of HNTG buffer was incubated with 20 μL of Protein G agarose (Thermo Fisher) for 1 h at 4 °C for preclearing. The cleared extract was collected by centrifugation and incubated with 2 μg of anti‐mTOR (H‐266) antibody from Santa Cruz Biotechnology (Dallas, TX, USA) overnight at 4 °C, followed by incubation with 20 μL of Protein G agarose for 3 h at 4 °C. The agarose beads were washed four times with HNTG buffer, and protein complexes were eluted with 2× SDS sample buffer by heating 95 °C for 3 min and used for western blotting.

### 2.8 Cap‐binding assay

Cells were lysed in NP‐40 lysis buffer (50 mm Tris/HCl, pH 7.5, 1% Nonidet P40, 150 mm NaCl) supplemented with proteinase and phosphatase inhibitors and cleared by centrifugation. The protein extracts (100 μg) were adjusted to 0.4 mL with NP‐40 lysis buffer and incubated with 20 μL of immobilized γ‐aminophenyl‐7‐methylguanosine (m^7^GTP)‐Agarose (Jena Bioscience, Jena, Germany) at 4 °C for 3 h. Agarose beads were washed three times with NP‐40 lysis buffer, and protein complexes were eluted with 2× SDS sample buffer and used for western blotting.

### 2.9 Quantification of cap‐dependent translation

Bicistronic luciferase reporter vector, pcDNA3‐rLuc‐PolioIRES‐fLuc, was generously provided by J. Blenis (Harvard Medical School). Cells treated with siRNA for 48 h were further transfected with a bicistronic luciferase reporter vector (0.5 μg) using LT1 reagent for 24 h. After lysis of the cells, luciferase activity was measured with a Luminometer (Promega, Madison, WI, USA) using a Dual‐Luciferase Reporter Assay System (Promega). The activity of cap‐dependent translation is defined as the ratio of *Renilla* luciferase to firefly luciferase activity.

### 2.10 Polysome profiling and analysis of mRNA distribution

The protocol for polysome fractionation to analyze mRNA distribution profiles was described previously (Panda et al., 2017). Briefly, HCT116 cells in 150‐mm dish were treated with 100 μg·mL^−1^ of cycloheximide (CHX) for 10 min, trypsinized for 3 min, and washed cells twice with cold PBS, and cell pellets were stored at −80 °C. All reagents were supplemented with 100 μg·mL^−1^ of CHX. Cells were lysed with 1 mL of extraction buffer [20 mm HEPES (pH 7.4), 150 mm KCl, 5 mm MgCl_2_, 1 mm DTT, 100 μg·mL^−1^ of CHX, 1% NP‐40] supplemented with protease inhibitor and 0.2 U·μL^−1^ RNaseOUT (Thermo Fisher). Equal amount of cleared cell extract was loaded onto top of 10‐50% sucrose gradient made with polysome buffer [20 mm HEPES (pH 7.4), 150 mm KCl, 5 mm MgCl_2_, 1 mm DTT, 100 μg·mL^−1^ of CHX] and centrifuged for 2 h in a SW41Ti rotor at 4 °C. Fractions (0.5 mL) in tubes from the top to the very bottom of the gradient were collected using the gradient fractionator while measuring absorbance at 254 nm. RNA was purified from the 0.5 mL pooled fractions using TRIzol (Thermo Fisher), and reverse transcription, real‐time PCR, and quantification of targets were performed as described previously (Kim et al., 2011). The sequence of primers for PCR is shown in Table S2.

### 2.11 ChIP assay

ChIP assays were performed using an EZ‐ChIP™ kit, according to the manufacturer's instructions (EMD Millipore, Darmstadt, Germany). Briefly, cells were treated with 1% formaldehyde for 10 min at room temperature and the reaction was quenched for 5 min in 1× glycine. The cells were washed with PBS, scraped, pelleted, and resuspended in SDS lysis buffer for sonication. The DNA was sheared with 10 rounds of 10‐s pulses followed by 1 min of rest on wet ice using a sonicator (Branson Digital Sonifier 450, Danbury, CT, USA) equipped with a 2‐mm tip and set to 30% of maximum power. Sonicated cell lysates were precipitated with 2 μg of STAT3 (C‐20) or normal rabbit IgG (Santa Cruz Biotechnology). The precipitated protein–DNA complexes were subjected to proteinase treatment, and the amount of DNA was determined by quantitative PCR. The primer sequences to confirm the binding of STAT3 to the promoter region of *MLST8* gene were 5′‐tgggctcagtgggatgtcct‐3′ (sense) and 5′‐aagctgcggctttctctcc‐3′ (antisense).

### 2.12 Reverse transcription and real‐time PCR

Total RNA preparation, reverse transcription, real‐time PCR, and quantification of targets were performed as described previously (Kim et al., 2011). The sequence of primers for PCR is shown in Table S2.

### 2.13 Promoter assay

The 316‐bp promoter region (−1201 to −894; +1, translation initiation site) of *MLST8* promoter was synthesized (Cosmo Genetech, Seoul, Korea) and subcloned to pGL3‐promoter firefly luciferase reporter (Promega). The −1201 to −951 and the −951 to −894 *MLST8* promoter regions were PCR‐amplified and subcloned to the firefly luciferase reporter vector. For promoter assay, 2 × 10^4^ cells were seeded 18 h before siRNA treatment in 24‐well plates. The reporter plasmids were transfected 48 h after siRNA treatment, and the cells were further incubated for 24 h before harvest. Transfection efficiency was normalized by cotransfection with a pCMV3.1‐*Renilla* vector in which *Renilla* luciferase coding region from pRL‐TK *Renilla* luciferase vector (Promega) was subcloned into pcDNA3.1 vector. Both the firefly luciferase and *Renilla* luciferase activities were quantified using a dual‐luciferase reporter assay system (Promega).

### 2.14 Statistical analysis

Experimental groups were compared with two‐tailed Student's unpaired *t*‐test, one‐way ANOVA with Newman–Keuls multiple comparison test, or two‐way ANOVA with two‐stage step‐up method of Benjamini, Krieger, and Yekutieli using the graphpad prism program (San Diego, CA, USA). Statistically significant differences are marked. *P* < 0.05 was considered statistically significant.

## 3 Results

### 3.1 *STAT3* knockdown decreases cap‐dependent translation in human cancer cells

**Fig. 1:**
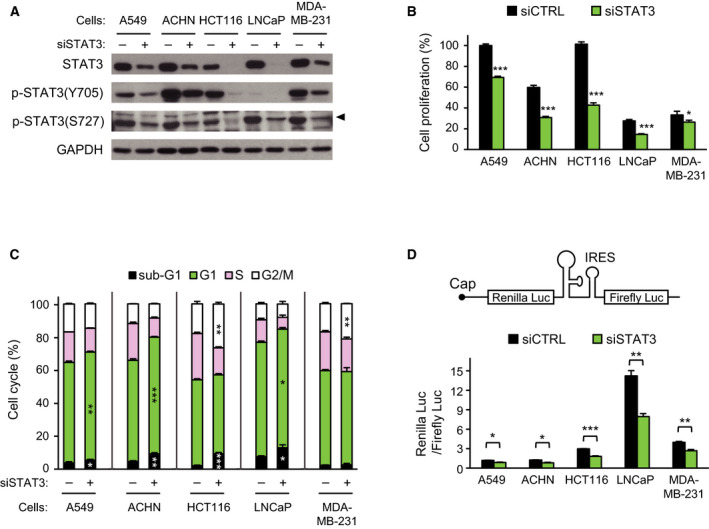
Inhibitory effect of *STAT3* knockdown on cell proliferation and cap‐dependent translation. Cancer cells were treated with 5 nm of siCTRL (−) or siSTAT3 (+) for 72 h. (A) Equal amounts of extracts were analyzed by western blotting with the antibodies indicated. (B) Relative cell proliferation of each group measured by WST‐1 assay was compared with the siCTRL group of A549 (*n* = 4). (C) Cell cycle distribution was analyzed with FACS (*n* = 3). (D) Diagram of bicistronic luciferase reporter is shown (top). Luciferase activities were measured by a dual‐luciferase assay, and the Renilla/firefly luciferase luminescence ratio was calculated for cap‐dependent translational activity (*n* = 4). Data are presented as mean ± SEM. Statistically significant differences are marked with *P < 0.05, **P < 0.01, and ***P < 0.001, respectively (*t*‐test).

Similar to the antiproliferative effect of *STAT3* knockdown on various cancer cells, *STAT3* siRNA treatment of HCT116 or MDA‐MB‐231 cells led to a decrease in cell proliferation (Fig. S1A). The level of STAT3 protein started to decrease gradually 24 h after *STAT3* siRNA treatment and dropped to < 20% of the level of control siRNA 72 h after treatment (Fig. S1B). We analyzed cell proliferation in other human cancer cells 72 h after siRNA treatment. Treatment with *STAT3* siRNA led to a decrease in the amount of both STAT3 protein and phospho‐STAT3 levels (Fig. 1A), and inhibition of cell proliferation (Fig. 1B). Cell cycle analysis revealed that *STAT3* knockdown caused increases in the number of cells in sub‐G1 phase, indicating the occurrence of cell death, in the majority of cell lines, except MDA‐MB‐231 cells. G1 phase arrest was also manifested in A549, ACHN, and LNCaP cells, in contrast to the G2/M phase arrest in HCT116 and MDA‐MB‐231 cells (Fig. 1C). These results demonstrated that *STAT3* knockdown induces either G1 or G2/M phase arrest, generally accompanied by cell death in human cancer cells.

Oncogenic AKT and ERK signaling converges on the activation of cap‐dependent translation, which is responsible for tumor cell growth (She et al., 2010). Cell cycle arrest and death caused by *STAT3* knockdown suggest that STAT3 may be involved in the regulation of cap‐dependent translation. Bicistronic luciferase reporter assays to measure cap‐dependent translation activity showed that *STAT3* knockdown reduced cap‐dependent translation in all cell lines tested (Fig. 1D), indicating cross‐talk between STAT3 and cap‐dependent translation‐related proteins.

### 3.2 Reduction in cap‐dependent translation by *STAT3* knockdown is correlated with the dephosphorylation of 4E‐BP1

**Fig. 2:**
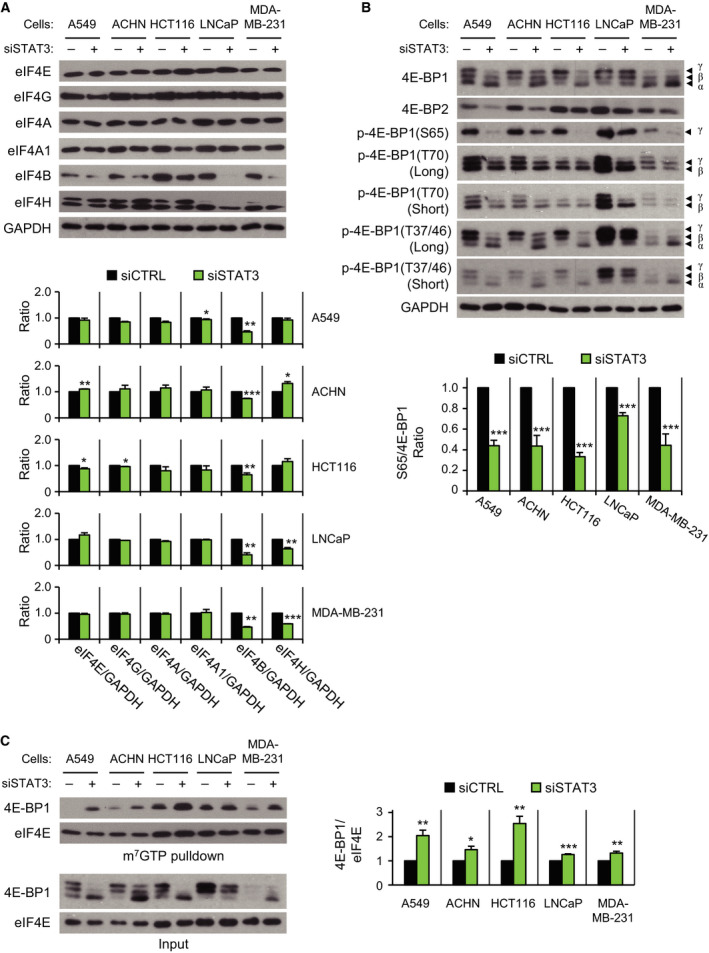
*STAT3* knockdown‐induced change in factors related to cap‐dependent translation initiation. Cancer cells were treated with 5 nm of siCTRL (−) or siSTAT3 (+) for 72 h. (A, B) Western blotting was performed using indicated antibodies (top). The band intensity of siSTAT3 group was normalized to that of siCTRL group in each cell line (bottom; *n* = 2–6). Long, long exposure; Short, short exposure. (C) Cell lysates were precipitated with m^7^GTP agarose beads, and eluted complexes were analyzed by western blotting with the antibodies indicated (left). The relative intensity of 4E‐BP1 to eIF4E of siSTAT3 group was compared with that of siCTRL group in each cell line (right; *n* = 4). Data are presented as mean ± SEM. Statistically significant differences are marked with *P < 0.05, **P < 0.01, and ***P < 0.001, respectively (*t*‐test).

Downregulation of cap‐dependent translation by *STAT3* knockdown could be due to the decreased expression of cap‐dependent translation initiation factors. We therefore examined the levels of proteins involved in cap‐dependent translation initiation (Fig. 2A). The majority of eIF4 family members exhibited constant or increased levels following *STAT3* knockdown. The level of eIF4B was reduced in every cell, but eIF4H levels were reduced only in LNCaP and MDA‐MB‐231 cells. In cap‐dependent translation initiation, eIF4B is known to enhance both ATPase and the helicase activities of eIF4A (Raught et al., 2004; Shahbazian et al., 2006). We then analyzed the effect of *EIF4B* knockdown on cap‐dependent translation. The level of eIF4B was downregulated by *EIF4B* siRNA treatment (Fig. S2A). However, only ACHN cells exhibited a decrease in cap‐dependent translation (Fig. S2B) and proliferation (Fig. S2C). Cell cycle analysis revealed that antiproliferation by *EIF4B* knockdown was mainly due to G1 phase arrest with a little increase in cell death (Fig. S2D). In all cells observed, the level of *EIF4B* mRNA was not decreased by siSTAT3 treatment (Fig. S2E). In contrast to the broad effect of *STAT3* knockdown on cell proliferation, cell cycle arrest, cell death, and cap‐dependent translation, the effect of *EIF4B* knockdown on cells was restricted to ACHN cells.

An increase in 4E‐BP1 protein also inhibits cap‐dependent translation (Richter and Sonenberg, 2005). However, 4E‐BP1 and 4E‐BP2 protein levels were either constant or downregulated by *STAT3* knockdown (Fig. 2B), indicating that the *STAT3* knockdown‐induced decrease in cap‐dependent translation is irrespective of 4E‐BP1 or 4E‐BP2 protein levels. *STAT3* knockdown led to shifts in 4E‐BP1 isoforms, which are generated by differential phosphorylation. It is well established that dissociation of 4E‐BP1 from the eIF4E/4E‐BP1 complex by hyperphosphorylation of 4E‐BP1 is a requisite for the activation of cap‐dependent translation (Richter and Sonenberg, 2005). Western blotting with phospho‐specific 4E‐BP1 antibody revealed reduced phosphorylation of 4E‐BP1 in every cell line by *STAT3* knockdown (Fig. 2B). To determine the amount of 4E‐BP1 associated with eIF4E, eIF4E protein was affinity‐purified with agarose beads coupled to the m^7^GTP, which mimics the mRNA 5′ cap structure (Sonenberg et al., 1978). The amount of 4E‐BP1 interacting with the m^7^GTP‐bound eIF4E was increased in all cell lines by *STAT3* knockdown (Fig. 2C), suggesting that dephosphorylation of 4E‐BP1, rather than the expression of cap‐dependent translation initiation factors, is directly correlated with the suppression of cap‐dependent translation by *STAT3* knockdown.

### 3.3 STAT3 regulates 4E‐BP1 phosphorylation

**Fig. 3:**
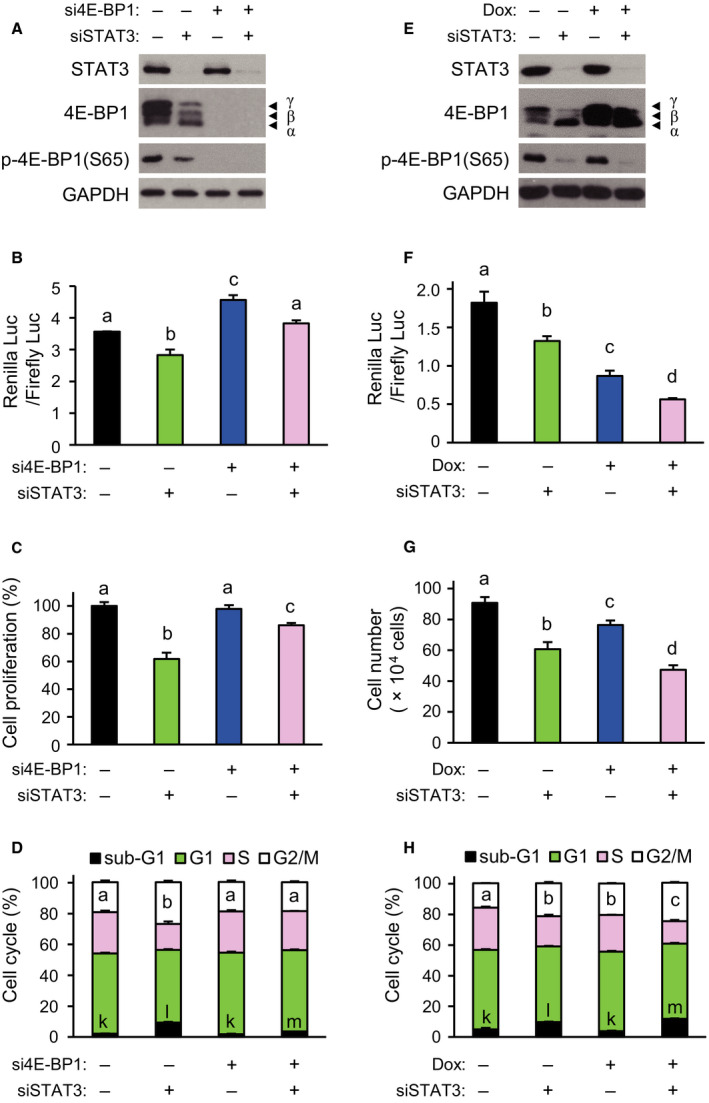
STAT3‐dependent modulation of 4E‐BP1 phosphorylation in HCT116 cells. In A to D, cancer cells were treated with 5 nm of siSTAT3 or 2 nm of si4EBP1 for 72 h (−, treatment with siCTRL; +, siSTAT3 or si4EBP1). (A) Western blotting was performed using equal amounts of extracts with the antibodies indicated. (B) The Renilla/firefly luciferase luminescence ratio was calculated for cap‐dependent translational activity (*n* = 3). (C) Cell proliferation was measured by WST‐1 assay (*n* = 3). (D) Cell cycle distribution was analyzed with FACS. (E–H) Cells were transfected with siSTAT3, and the cells were further transfected 24 h after siRNA transfection with a bicistronic luciferase reporter and 4E‐BP1 dominant‐active mutant vector, followed by doxycycline treatment for 48 h before harvesting cells. (E) Western blotting was performed using indicated antibodies. (F) The Renilla/firefly luciferase luminescence ratio was calculated for cap‐dependent translational activity (*n* = 3). (G) Counting of viable cells (*n* = 3). (H) Cell cycle distribution was analyzed by FACS (*n* = 3). Data are presented as mean ± SEM. Bars (B–D and F–H) with different letters are significantly different (*P* < 0.05), one‐way ANOVA. For D and H, a–c for G2/M; k–m for sub‐G1, respectively.

Similar to the hyperphosphorylation of 4E‐BP1, a decrease in 4E‐BP1 protein level resulted in reduction in the interaction between eIF4E and 4E‐BP1 and the subsequent activation of cap‐dependent translation (Connolly et al., 2006; She et al., 2010). To further test whether a decrease in 4E‐BP1 may alleviate the effect of *STAT3* knockdown on cells, *4EBP1* siRNA and *STAT3* siRNA were treated together in HCT116 cells. Treatment with *4EBP1* siRNA showed near‐complete knockdown of 4EBP1 and phospho‐4E‐BP1 levels (Fig. 3A). *4EBP1* knockdown abolished the downregulation of cap‐dependent translation induced by *STAT3* knockdown (Fig. 3B). Moreover, the inhibition of cell proliferation was much less when *STAT3* and *4EBP1* were both knocked down, than when *STAT3* was knocked down alone (Fig. 3C). Cell cycle analysis showed that cotreatment with *4EBP1* siRNA led to the complete disappearance of G2/M phase arrest, and significant recovery of sub‐G1 phase resulted from *STAT3* knockdown alone (Fig. 3D).

To test whether further inhibition of 4E‐BP1 phosphorylation aggravated the effect of *STAT3* knockdown on cells, HCT116 cells were cotreated with *STAT3* siRNA and doxycycline‐dependent mutant 4E‐BP1 expression vector, replacing four phosphorylation sites (T37, T46, S65, and T70) with alanine to maintain the constitutive binding of mutant 4E‐BP1 to eIF4E (Rong et al., 2008). Mutant 4E‐BP1 expression alone did not cause the downregulation of phospho‐4E‐BP1 level, but it further decreased the phospho‐4E‐BP1(S65) level induced by *STAT3* knockdown (Fig. 3E). Cotreatments resulted in more suppression of cap‐dependent translation than treatment with mutant 4E‐BP1 vector or *STAT3* siRNA alone (Fig. 3F). Similarly, inhibition of cell proliferation was much greater by the cotreatments compared with either treatment alone (Fig. 3G). Cell cycle analysis showed that inhibition of cell proliferation was due to increases in both G2/M and sub‐G1 phases by the cotreatments (Fig. 3H). These data support the hypothesis that a reduction in cap‐dependent translation by *STAT3* knockdown is mediated by the dephosphorylation of 4E‐BP1 in cancer cells.

**Fig. 4:**
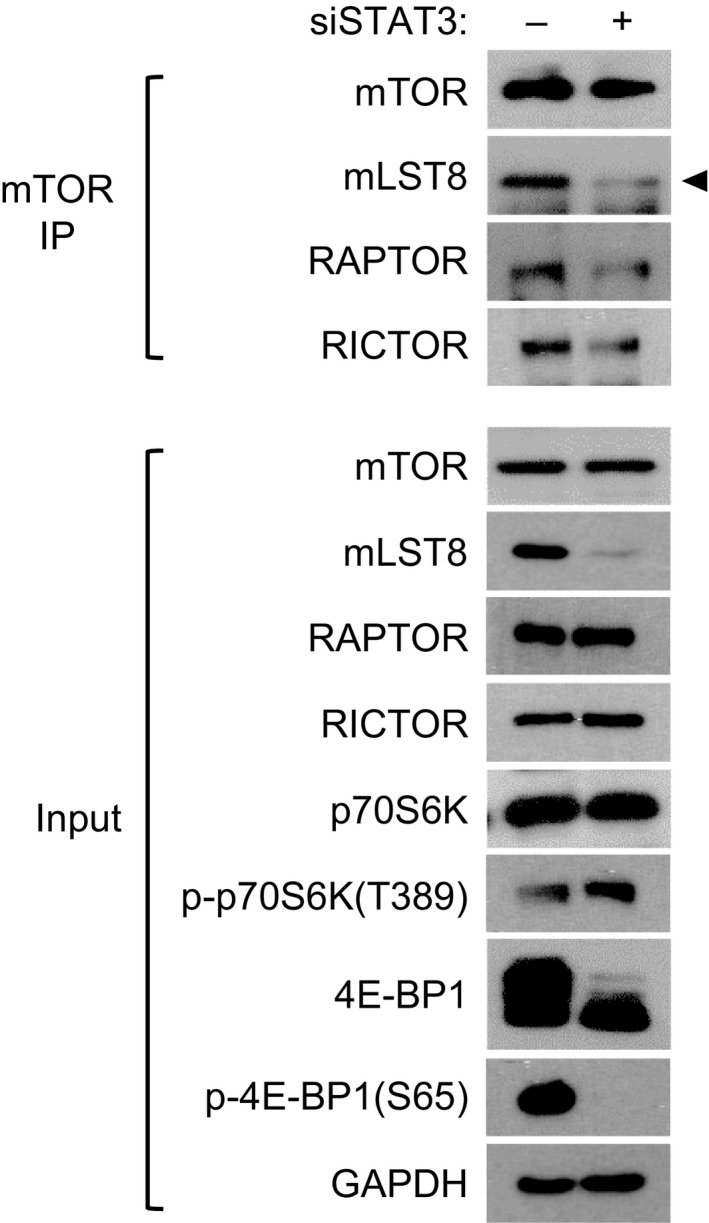
Correlation of *STAT3* knockdown‐induced 4E‐BP1 dephosphorylation and downregulation of mTORC1. HCT116 cells were transfected with 5 nm of siSTAT3 or siCTRL for 72 h. Cell lysates were immunoprecipitated with mTOR antibody, followed by western blotting with the indicated antibodies using IP and input samples. Representative images of three independent experiments are shown.

We also analyzed the effects of *STAT3* knockdown on the formation of mTORC1 to find a reason for the observed decrease in 4E‐BP1 phosphorylation. Immunoprecipitation (IP) with mTOR antibody revealed that *STAT3* knockdown reduced the interaction of mLST8 and Raptor with mTOR (Fig. 4). Western blot analysis of the proteins showed that *STAT3* knockdown reduced the total amount of mLST8 but not Raptor. In contrast to the dephosphorylation of the 4E‐BP1, phospho‐p70S6K, another substrate for mTORC1, was increased slightly by *STAT3* knockdown. These results suggest that the STAT3‐mediated *MLST8* gene expression is likely to be involved in the interaction of mTOR and Raptor, and subsequent phosphorylation of 4E‐BP1.

### 3.4 mLST8 mediates STAT3‐dependent 4E‐BP1 phosphorylation

T37, T46, S65, and T70 of 4E‐BP1 are phosphorylated by mTORC1, and activation of mTORC1 is controlled by regulatory proteins associated directly with mTOR or indirectly with mTORC1 (Hay and Sonenberg, 2004). Hence, we hypothesized that STAT3 mediates 4E‐BP1 phosphorylation through regulation of the genes for mTORC1 components, especially mLST8. To test this hypothesis, we measured the mRNA levels of mTORC1 components and related proteins after *STAT3* knockdown. The mRNA levels of *DEPTOR*, *PDIA3*, *PRAS40, RAC1, RAPTOR*, and *TTI1* were consistent in every cell line, whereas the level of *MLST8* mRNA was downregulated by *STAT3* knockdown in all of the cell lines, and the level of *TELO2* was also attenuated in most cell lines, except MDA‐MB‐231 cells (Fig. S3).

**Fig. 5:**
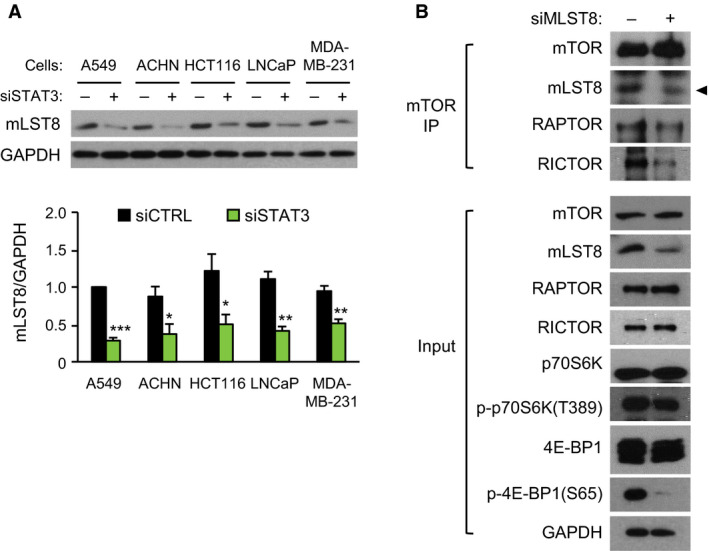
Intermediation of mLST8 in STAT3‐dependent 4E‐BP1 phosphorylation. (A) Western blotting (top) and mLST8 protein level were quantified at 72 h after 5 nm of siRNA treatment in human cancer cells. The relative intensity of mLST8 to GAPDH was normalized to that of siCTRL group of A549 (bottom; *n* = 3). Data are presented as mean ± SEM. Statistically significant differences are marked with *P < 0.05, **P < 0.01, and ***P < 0.001, respectively (*t*‐test). (B) HCT116 cells were transfected with 5 nm of siCTRL or siMLST8 for 24 h. Equal amounts of HCT116 cell lysates were immunoprecipitated with mTOR antibody. The proteins in the immunoprecipitated and input were analyzed with western blotting using the antibodies indicated. Representative images of three independent experiments are shown.

Attenuation of *MLST8* mRNA levels by *STAT3* knockdown resulted in downregulation of mLST8 protein levels (Fig. 5A). IP with mTOR antibody revealed that *MLST8* knockdown decreased the interaction of mLST8 and Raptor with mTOR, although western blot analysis of the protein showed no changes in mTOR or Raptor following *MLST8* knockdown (Fig. 5B). *MLST8* knockdown resulted in a marked reduction in 4E‐BP1 phosphorylation similar to those of *STAT3* knockdown, but the level of phospho‐p70S6K was not changed (Fig. 5B).

**Fig. 6:**
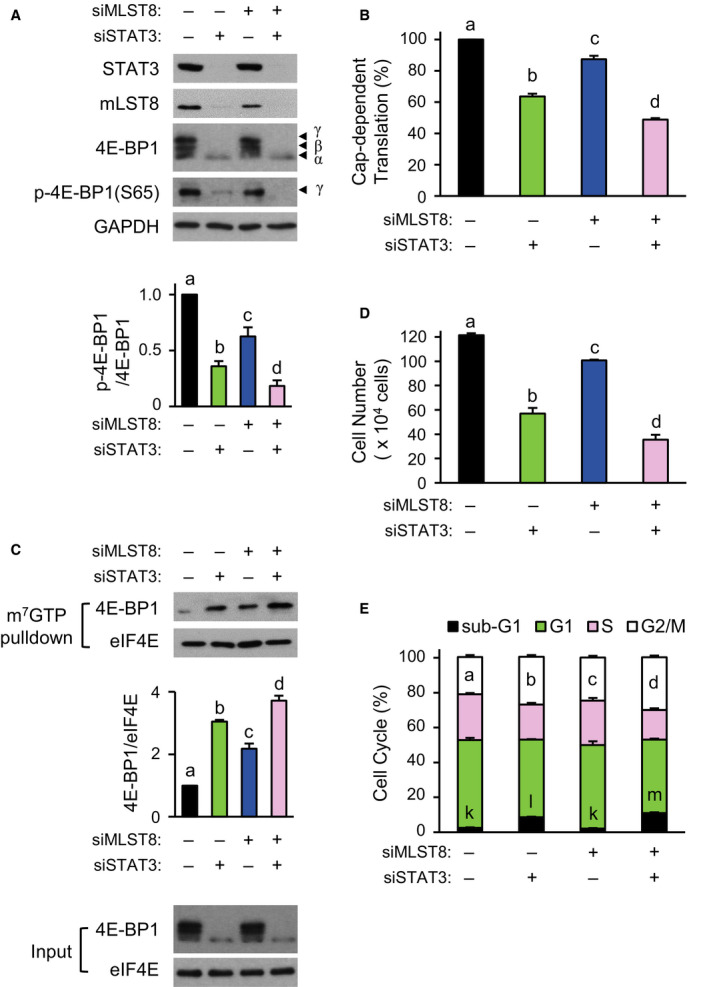
Added effect of *STAT3* and *MLST8* knockdown on cap‐dependent translation in HCT116 cells. siSTAT3 (5 nm) were transfected into cells for 48 h. The cells were then transfected with siMLST8 (1 nm) for 24 h. (A) Western blotting was performed using equal amounts of extracts with the antibodies indicated (top), and the band intensity of phospho‐4E‐BP1(S65; bottom) was quantified (*n* = 3). (B) siSTAT3‐transfected cells were further transfected with siMLST8 and bicistronic luciferase reporter to measure luciferase activities. Cap‐dependent translational activity of each group was compared with siCTRL group (*n* = 3). (C) m^7^GTP pull‐down assay was performed after serial treatment with siSTAT3 and siMLST8 (top), and the cap‐binding 4E‐BP1 index was determined by the ratio of 4E‐BP1 to eIF4E (bottom; *n* = 3). (D) Counting of cell numbers (*n* = 3). (E) Cell cycle distribution was analyzed by FACS (*n* = 3). Data are presented as mean ± SEM. Different letters (A–E) are significantly different (*P* < 0.05), one‐way ANOVA. For E, a–d for G2/M; k–m for sub‐G1, respectively.

To investigate the significance of mLST8 downregulation by *STAT3* knockdown, HCT116 cells were treated with *MLST8* siRNA alone or with *MLST8* siRNA and *STAT3* siRNA together. *STAT3* knockdown‐induced reduction in mLST8 protein levels was further reinforced by treatment with *MLST8* siRNA (Fig. 6A). Although *MLST8* knockdown was sufficient to attenuate phospho‐4E‐BP1(S65) level, co‐knockdown led to more attenuation than either *STAT3* or *MLST8* knockdown (Fig. 6A). Cap‐dependent translation was reduced by *MLST8* knockdown and was further suppressed by co‐knockdown of *MLST8* and *STAT3* (Fig. 6B). The amount of 4E‐BP1 interacting with m^7^GTP‐bound eIF4E was increased by *MLST8* knockdown and was further increased by co‐knockdown (Fig. 6C). *MLST8* or *STAT3* knockdown resulted in marked reduction in the phosphorylation of AKT(S473), but no reduction in phospho‐p70S6K or phospho‐AKT(T308) levels (Fig. S4A). Inhibition of cell proliferation reflected the extent of cap‐dependent translation (Fig. 6D). Co‐knockdown increased the numbers of cells in the G2/M and sub‐G1 phases (Fig. 6E). These results indicate that *STAT3* knockdown‐induced decrease in mLST8 level mediates the downregulation of 4E‐BP1 phosphorylation and subsequent decrease in cap‐dependent translation and cell proliferation.

### 3.5 mLST8 overexpression compensates for reduction in *STAT3* knockdown‐induced cap‐dependent translation

**Fig. 7:**
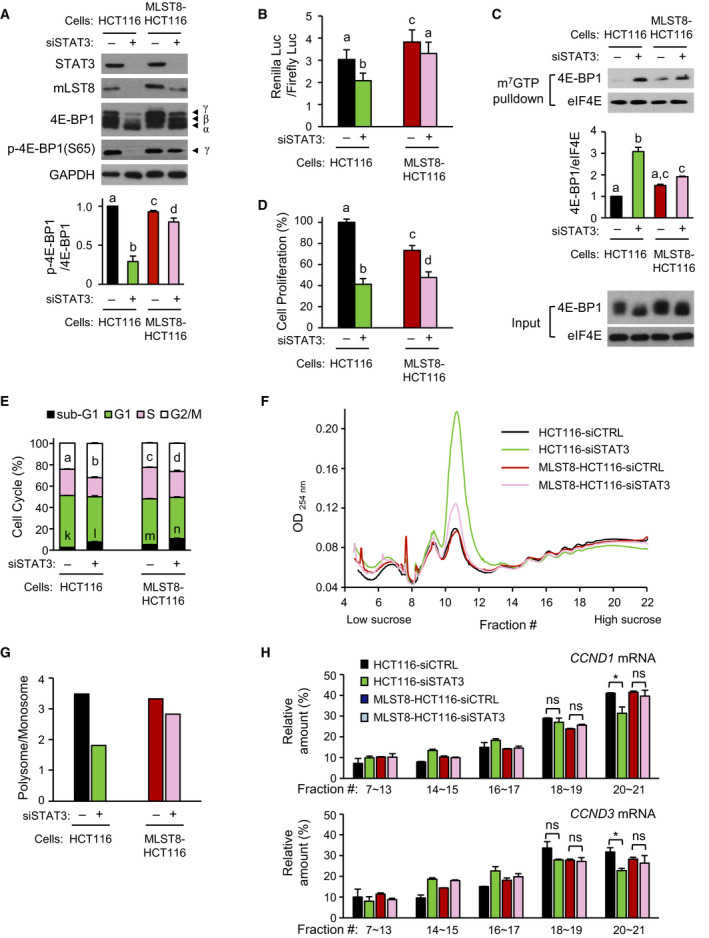
Amelioration of cap‐dependent translation in mLST8‐overexpressed HCT116 cells. (A) siSTAT3 were transfected into control HCT116 and MLST8‐HCT116 cells for 72 h. Western blotting was performed using equal amounts of extracts with indicated antibodies, and the band intensity of phospho‐4E‐BP1(S65; bottom) was quantified (*n* = 4). (B) siSTAT3 were transfected into cells for 48 h followed by secondary transfection of cells with the bicistronic luciferase reporter for 24 h. Cap‐dependent translational activity of each group was compared with siCTRL group using dual‐luciferase assay (*n* = 4). (C) m^7^GTP pull‐down extract was detected by western blotting using indicated antibodies, and the cap‐binding 4E‐BP1 index was determined by the ratio of 4E‐BP1 to eIF4E (bottom; *n* = 3). (D) Relative cell proliferation was determined by counting the viable cells (*n* = 3). (E) Cell cycle distribution was analyzed by FACS (*n* = 3). (F) Cell extract was obtained after 48 h of treatment with 2 nm siRNA, and polysome fractionation was performed. Elution profile was shown by reading absorbance readings at 254 nm. (G) Relative polysome‐to‐monosome ratios in F are shown. (H) RNA was extracted by pooling each fraction as indicated (#7–#13, monosome; #14–#21, polysome), and relative amounts of mRNA in fractions were expressed when the total fractions of *CCND1* and *CCND3* mRNA were 100% using qRT–PCR (*n* = 2). The RNA difference in each fraction was normalized using the value of RNA polymerase II (*POLR2A*) mRNA. Data are presented as mean ± SEM. Statistically significant differences are marked with different letters (A–E; for E, a–d for G2/M; k–n for sub‐G1; *P* < 0.05) or with *P < 0.05; ns, statistically insignificant, two‐way ANOVA.

A stable HCT116 cell line (MLST8‐HCT116) overexpressing mLST8 was generated to supplement the decrease in mLST8 level caused by *STAT3* knockdown. Overexpression of mLST8 prevented the *STAT3* knockdown‐induced decrease in phospho‐4E‐BP1(S65) level (Fig. 7A). Overexpression of mLST8 partially diminished the *STAT3* knockdown‐induced downregulation of cap‐dependent translation (Fig. 7B), which correlated well with the amount of 4E‐BP1 interacting with m^7^GTP‐bound eIF4E (Fig. 7C). *STAT3* knockdown‐induced decrease in the phosphorylation of AKT(S473) did not occur in the MLST8‐HCT116 cells (Fig. S4B). *STAT3* knockdown‐induced inhibition of cell proliferation was also partially restored by mLST8 overexpression (Fig. 7D), reflecting partial recovery from G2/M phase arrest, but not from cell death (Fig. 7E).

Polysomal profiling was performed by *STAT3* knockdown in HCT116 cells and MLST8‐HCT116 cells (Fig. 7F). In HCT116 cells, the monosome (near Fraction #11) portion was significantly increased by *STAT3* knockdown, but in MLST8‐HCT116 cells, the monosome portion was less increased. Conversely, in the HCT116 cells, the polysome (Fractions #17–#21) portion was reduced by *STAT3* knockdown, but in the MLST8‐HCT116 cells, the reduction in the polysome portion was minimal. Overall, the polysome‐to‐monosome ratio was dramatically reduced by *STAT3* knockdown in HCT116 cells, but this ratio was alleviated by mLST8 overexpression (Fig. 7G). RNA was extracted from polysome fractions (#7–#13, monosome; #14–#21, low‐molecular‐weight to high‐molecular‐weight polysome), and the relative amount of *CCND1* or *CCND3* mRNA, two mRNAs translationally controlled by the mTORC1/4E‐BP/eIF4E axis, was examined in the fractions using quantitative reverse transcription and real‐time PCR (qRT–PCR; Fig. 7G). The ratio of high‐molecular‐weight polysome of *CCND1* and *CCND3* mRNA (Fractions #20 to #21) was reduced in HCT116 cells by *STAT3* knockdown but not in MLST8‐HCT116 cells. Overall, these results suggest that cap‐dependent translation is reduced by *STAT3* knockdown and can be rescued partially by mLST8 overexpression.

### 3.6 STAT3 acts as a transcription factor for the *MLST8* gene expression

**Fig. 8:**
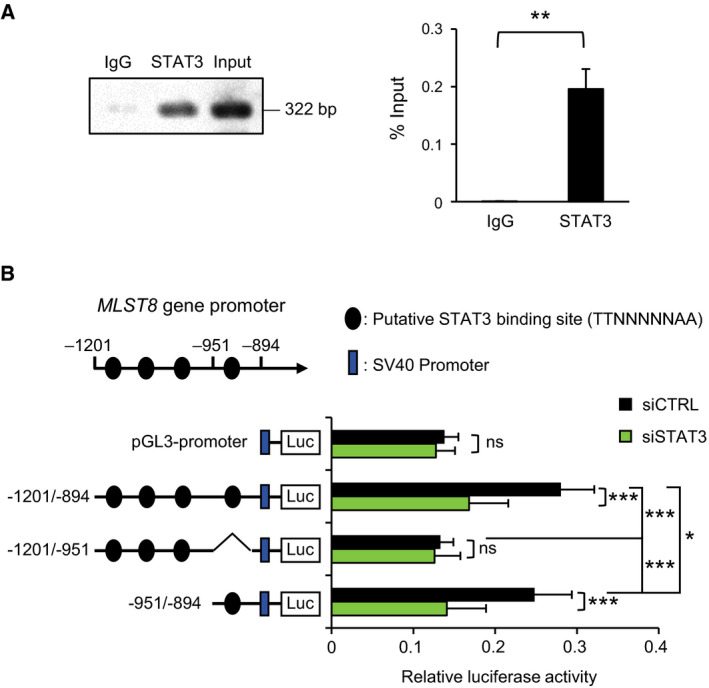
Identification of STAT3‐binding sites in *MLST8* promoter. (A) Protein–DNA complexes from HCT116 cells were precipitated with either STAT3 antibody or normal IgG and the amount of DNA was determined by PCR with primers for promoter regions of the *MLST8* gene, followed by agarose electrophoresis (left) or quantitative PCR (right; *n* = 3). (B) Luciferase reporter assay was performed in HCT116 cells treated with 5 nm of siCTRL or siSTAT3. *MLST8* promoter‐luciferase constructs and pCMV3.1‐*Renilla* vector were transfected 48 h later, and the cells were further incubated for 24 h before harvest. Firefly luciferase activity was normalized with *Renilla* luciferase activity (*n* = 4). Data are presented as mean ± SEM. Statistically significant differences are marked with *P < 0.05, **P < 0.01, and ***P < 0.001, respectively; ns, statistically insignificant (A, *t*‐test; B, two‐way ANOVA).

Because STAT3 is a well‐known transcription factor, we hypothesized that STAT3 acts as a transcription factor for *MLST8* gene expression. We found that the −1201 to −894 region of the *MLST8* promoter contains four potential STAT3‐binding sites (TTN_5_AA). ChIP assays using STAT3 antibody in HCT116 cells revealed that STAT3 interacted with the promoter region including the −1201 to −894 region of the *MLST8* gene (Fig. 8A). Promoter mapping with luciferase reporter assays showed that the downregulation of *MLST8* gene expression induced by *STAT3* knockdown was mediated by the −951 to −894 but not by the −1201 to −951 region (Fig. 8B). These results indicated that in the −951 to −894 region of the *MLST8* promoter is likely to include the STAT3‐binding site.

JAK/STAT3 activation by IL‐6 plays an important role in STAT3 target gene expression (Johnson et al., 2018), and we investigated whether *MLST8* gene expression is regulated by IL‐6 treatment in HCT116 cells (Fig. S5). Western blotting showed that phospho‐STAT3(Y705) was increased by IL‐6 treatment, and the expression of the IL‐6‐inducing gene *CYP1B1* mRNA was increased as a result. However, the expression of *MLST8* did not increase at the mRNA or protein level. Since STAT3 can form heterodimer with STAT1, the effect of STAT1 on *MLST8* gene expression was also investigated in HCT116 cells. The levels of phospho‐STAT1(Y701) and phospho‐STAT3(Y705) decreased, but the expression of *MLST8* was unchanged by *STAT1* knockdown at the mRNA or protein level (Fig. S6). *MLST8* mRNA expression is not affected by the phosphorylation status of STAT3(Y705), suggesting that it is expected to be regulated through unphosphorylated STAT3 as described elsewhere (Srivastava and DiGiovanni, 2016).

## 4 Discussion

STAT3 binds to consensus cis‐acting elements of target genes in the nucleus, thus driving the transcription of a variety of genes encoding regulators of growth (such as cyclin D1 and c‐Myc), survival (such as BCL‐xL and survivin), angiogenesis (such as HIF1‐α and VEGF), metastasis (such as MMP1 and vimentin) of cancer cells, and cancer immune evasion (such as IL‐6 and TGF‐β) (Carpenter and Lo, 2014). In cancers, hyperactivity of mTORC1/2 overlapped with the function of STAT3 especially in cell growth, survival, and migration (Blenis, 2017; Saxton and Sabatini, 2017), where STAT3 phosphorylation by mTORC1 is probably involved. mTORC1 is one of the kinases that can phosphorylate STAT3 at S727, resulting in maximal activation of STAT3‐responsive reporter (Yokogami et al., 2000), but the details of the regulation of the mTOR pathway by STAT3 are not known. Our results indicated that STAT3 regulates *MLST8* gene expression and facilitates the formation of mTORC1/2, cooperating with the mTOR pathway in cancer cell proliferation.

Because mLST8 is a core component of both mTORC1 and mTORC2 (Saxton and Sabatini, 2017), it is probable that mLST8 protein level reflects mTORC1/2 formation and kinase activity. The phosphorylation status of 4E‐BP1(T37/46, S65, T70) and AKT(S473), substrates of mTORC1 and mTORC2, respectively, that we observed using knockdown or overexpression of mLST8 in HCT116 cells was consistent with that previously reported (Kakumoto et al., 2015). *STAT3* knockdown‐induced downregulation of 4E‐BP1 and AKT phosphorylation observed in our study was correlated with mLST8 level, suggesting that STAT3 may regulate mTORC1/2 formation and kinase activity through *MLST8* gene expression. *STAT3* knockdown‐induced decreases in 4E‐BP1 and AKT phosphorylation levels were further attenuated by *MLST8* knockdown or recovered by mLST8 overexpression, indicating that mLST8 level is likely to be an important factor regulating mTORC1/2 kinase activity. Besides 4E‐BP1, p70S6K phosphorylation by mTORC1 plays an important role in translational initiation and elongation (Truitt and Ruggero, 2016). However, p70S6K(T389) phosphorylation was not reduced, but slightly increased by *STAT3* knockdown or unchanged by *MLST8* knockdown, similar to a previous report of *MLST8* knockdown in HCT116 cells (Kakumoto et al., 2015). Knockdown of *M* *TOR* reduced the phosphorylation of p70S6K and 4E‐BP1 in HCT116 cells (Zhang et al., 2009). In contrast, mTOR inhibitors reduced p70S6K phosphorylation but not 4E‐BP1 phosphorylation in HCT116 cells (Zhang and Zheng, 2012), indicating that the phosphorylation of p70S6K and 4E‐BP1 is independently regulated, depending on the situation. Both mTORC1‐mediated p70S6K and 4E‐BP1 phosphorylation may be initially reduced by mLST8 downregulation, but p70S6K phosphorylation status may be compensated by another kinase such as PDK1 (Balendran et al., 1999) or PI3K (Gonzalez‐Garcia et al., 2002), due to a heterozygous substitution mutation in PIK3CA H1047R, which produces constitutive activation of PI3K in HCT116 cells (Samuels et al., 2005). However, the detailed mechanisms underlying these phenomena need to be investigated in future studies.

Our results showed that eIF4B protein was regulated by STAT3 in a variety of cancer cells, and eIF4B downregulation itself had little effect on cap‐dependent translation. However, in cancer cells such as ACHN cells, in which eIF4B plays an important role, it is possible to stimulate cell growth by regulating cap‐dependent translation through the expression of eIF4B and mLST8 by STAT3. STAT3 phosphorylation by mTORC1 produces maximal transcriptional activation of STAT3‐responsive genes (Yokogami et al., 2000), and our results indicate that STAT3 regulates cap‐dependent translation by regulating 4E‐BP1 phosphorylation through *MLST8* transcription, suggesting a positive feedback regulation between STAT3 and mTORC1. Many STAT3 target genes related to cancer are also regulated at the translational level by cap‐dependent translation (Carpenter and Lo, 2014; De Benedetti and Graff, 2004; Musa et al., 2016). Thus, STAT3 target genes in cancer can be expressed very efficiently both at the transcription level and at the translation level. This phenomenon is comparable to the target gene expression control of c‐Myc, the well‐known transcription factor, in which the c‐Myc oncogene regulates the expression of eIF4F components, which in turn regulate *c‐Myc* mRNA translation, establishing a positive feedforward loop for deregulation of translational control and aberrant growth in cancer cells (Lin et al., 2009).

We also found that *STAT3* knockdown‐induced attenuation of cell proliferation resulted from cell death and cell cycle arrest, depending on cancer cell context. In HCT116 cells, cell death and G2/M phase arrest preferentially occurred following *STAT3* knockdown. The partial recovery of G2/M phase arrest by co‐knockdown of *4EBP1* and the additive attenuation of cell proliferation by the expression of mutant 4E‐BP1 suggests that STAT3‐dependent 4E‐BP1 phosphorylation is likely to play a role in cell cycle progression and cell death. In contrast, the inhibition of mTOR by siRNA induces cell death and G1 phase arrest in HCT116 cells (Gulhati et al., 2009; Zhang et al., 2009). This may be due to the differential effect of *STAT3* and *MTOR* knockdown on cell cycle arrest, since *MTOR* knockdown induces a decrease in p70S6K and 4E‐BP1 phosphorylation, whereas *STAT3* knockdown only induces a decrease in 4E‐BP1 phosphorylation. Therefore, it appears that the STAT3 and mTOR pathways are common factors in controlling cell death and the cell cycle, but there are mutually specific pathways.

## 5 Conclusions

In this work, we have demonstrated that STAT3 regulates *MLST8* gene expression, resulting in the facilitation of the formation of mTORC1, inducing phosphorylation of 4E‐BP1 and upregulation of cap‐dependent translation. STAT3 induces the expression of multiple genes related to cancer cell growth and survival, and also could activate cap‐dependent translation through *MLST8* expression, further promoting cancer cell proliferation. We suggest that the STAT3/mLST8/4E‐BP1 signal pathway might be a valuable target for cancer therapy.

## Conflict of interest

The authors declare no conflict of interest.

## Author contributions

HL, HC, and DL designed research; HL, HC, and HK performed experiments; HL, HJ, and DL analyzed data and wrote the manuscript.

## Supporting information


**Fig. S1.** Time‐course changes of cell viability and protein level after STAT3 knockdown.
**Fig. S2.** Effect of eIF4B knockdown on cap‐dependent translation.
**Fig. S3.** The mRNA expression of mTORC1 components in STAT3 knockdown cells.
**Fig. S4.** Effect of STAT3 knockdown on mTOR signaling in MLST8 knockdown or MLST8‐overexpressed HCT116 cells.
**Fig. S5.** Effect of IL‐6 treatment on MLST8 gene expression in HCT116 cells.
**Fig. S6.** Effect of STAT1 knockdown on MLST8 gene expression in HCT116 cells.
**Table S1.** The siRNA target sequences.
**Table S2.** The primer sequences for real‐time PCR.

## References

[mol212735-bib-0001] Balendran A , Currie R , Armstrong CG , Avruch J and Alessi DR (1999) Evidence that 3‐phosphoinositide‐dependent protein kinase‐1 mediates phosphorylation of p70 S6 kinase *in vivo* at Thr‐412 as well as Thr‐252. J Biol Chem 274, 37400–37406. 10601311 10.1074/jbc.274.52.37400

[mol212735-bib-0002] Blenis J (2017) TOR, the gateway to cellular metabolism, cell growth, and disease. Cell 171, 10–13. 28888322 10.1016/j.cell.2017.08.019

[mol212735-bib-0003] Cao HH , Tse AK , Kwan HY , Yu H , Cheng CY , Su T , Fong WF and Yu ZL (2014) Quercetin exerts anti‐melanoma activities and inhibits STAT3 signaling. Biochem Pharmacol 87, 424–434. 24275163 10.1016/j.bcp.2013.11.008

[mol212735-bib-0004] Carpenter RL and Lo HW (2014) STAT3 target genes relevant to human cancers. Cancers (Basel) 6, 897–925. 24743777 10.3390/cancers6020897 PMC4074809

[mol212735-bib-0005] Catena V and Fanciulli M (2017) Deptor: not only a mTOR inhibitor. J Exp Clin Cancer Res 36, 12. 28086984 10.1186/s13046-016-0484-y PMC5237168

[mol212735-bib-0006] Connolly E , Braunstein S , Formenti S and Schneider RJ (2006) Hypoxia inhibits protein synthesis through a 4E‐BP1 and elongation factor 2 kinase pathway controlled by mTOR and uncoupled in breast cancer cells. Mol Cell Biol 26, 3955–3965. 16648488 10.1128/MCB.26.10.3955-3965.2006 PMC1489005

[mol212735-bib-0007] Darnell JE Jr (1997) STATs and gene regulation. Science 277, 1630–1635. 9287210 10.1126/science.277.5332.1630

[mol212735-bib-0008] De Benedetti A and Graff JR (2004) eIF‐4E expression and its role in malignancies and metastases. Oncogene 23, 3189–3199. 15094768 10.1038/sj.onc.1207545

[mol212735-bib-0009] Evren S , Dermen A , Lockwood G , Fleshner N and Sweet J (2011) mTOR‐RAPTOR and 14‐3‐3sigma immunohistochemical expression in high grade prostatic intraepithelial neoplasia and prostatic adenocarcinomas: a tissue microarray study. J Clin Pathol 64, 683–688. 21653658 10.1136/jclinpath-2011-200083

[mol212735-bib-0010] Fuhler GM , Tyl MR , Olthof SG , Lyndsay Drayer A , Blom N and Vellenga E (2009) Distinct roles of the mTOR components Rictor and Raptor in MO7e megakaryocytic cells. Eur J Haematol 83, 235–245. 19341427 10.1111/j.1600-0609.2009.01263.x

[mol212735-bib-0011] Gonzalez‐Garcia A , Garrido E , Hernandez C , Alvarez B , Jimenez C , Cantrell DA , Pullen N and Carrera AC (2002) A new role for the p85‐phosphatidylinositol 3‐kinase regulatory subunit linking FRAP to p70 S6 kinase activation. J Biol Chem 277, 1500–1508. 11684675 10.1074/jbc.M103808200

[mol212735-bib-0012] Grabiner BC , Nardi V , Birsoy K , Possemato R , Shen K , Sinha S , Jordan A , Beck AH and Sabatini DM (2014) A diverse array of cancer‐associated MTOR mutations are hyperactivating and can predict rapamycin sensitivity. Cancer Discov 4, 554–563. 24631838 10.1158/2159-8290.CD-13-0929 PMC4012430

[mol212735-bib-0013] Gulhati P , Cai Q , Li J , Liu J , Rychahou PG , Qiu S , Lee EY , Silva SR , Bowen KA , Gao T *et al* (2009) Targeted inhibition of mammalian target of rapamycin signaling inhibits tumorigenesis of colorectal cancer. Clin Cancer Res 15, 7207–7216. 19934294 10.1158/1078-0432.CCR-09-1249 PMC2898570

[mol212735-bib-0014] Hay N and Sonenberg N (2004) Upstream and downstream of mTOR. Genes Dev 18, 1926–1945. 15314020 10.1101/gad.1212704

[mol212735-bib-0015] Huynh J , Etemadi N , Hollande F , Ernst M and Buchert M (2017) The JAK/STAT3 axis: a comprehensive drug target for solid malignancies. Semin Cancer Biol 45, 13–22. 28647610 10.1016/j.semcancer.2017.06.001

[mol212735-bib-0016] Johnson DE , O'Keefe RA and Grandis JR (2018) Targeting the IL‐6/JAK/STAT3 signalling axis in cancer. Nat Rev Clin Oncol 15, 234–248. 29405201 10.1038/nrclinonc.2018.8 PMC5858971

[mol212735-bib-0017] Kakumoto K , Ikeda J , Okada M , Morii E and Oneyama C (2015) mLST8 promotes mTOR‐mediated tumor progression. PLoS One 10, e0119015. 25906254 10.1371/journal.pone.0119015 PMC4408021

[mol212735-bib-0018] Kim DH , Sarbassov DD , Ali SM , King JE , Latek RR , Erdjument‐Bromage H , Tempst P and Sabatini DM (2002) mTOR interacts with raptor to form a nutrient‐sensitive complex that signals to the cell growth machinery. Cell 110, 163–175. 12150925 10.1016/s0092-8674(02)00808-5

[mol212735-bib-0019] Kim K , Lee H , Threadgill DW and Lee D (2011) Epiregulin‐dependent amphiregulin expression and ERBB2 signaling are involved in luteinizing hormone‐induced paracrine signaling pathways in mouse ovary. Biochem Biophys Res Commun 405, 319–324. 21237132 10.1016/j.bbrc.2011.01.039

[mol212735-bib-0020] Lee D , Yu M , Lee E , Kim H , Yang Y , Kim K , Pannicia C , Kurie JM and Threadgill DW (2009) Tumor‐specific apoptosis caused by deletion of the ERBB3 pseudo‐kinase in mouse intestinal epithelium. J Clin Invest 119, 2702–2713. 19690388 10.1172/JCI36435 PMC2735918

[mol212735-bib-0021] Lee H , Lee H , Chin H , Kim K and Lee D (2014) ERBB3 knockdown induces cell cycle arrest and activation of Bak and Bax‐dependent apoptosis in colon cancer cells. Oncotarget 5, 5138–5152. 24970817 10.18632/oncotarget.2094 PMC4148128

[mol212735-bib-0022] Lin CJ , Malina A and Pelletier J (2009) c‐Myc and eIF4F constitute a feedforward loop that regulates cell growth: implications for anticancer therapy. Cancer Res 69, 7491–7494. 19773439 10.1158/0008-5472.CAN-09-0813

[mol212735-bib-0023] Lin L , Liu A , Peng Z , Lin HJ , Li PK , Li C and Lin J (2011) STAT3 is necessary for proliferation and survival in colon cancer‐initiating cells. Cancer Res 71, 7226–7237. 21900397 10.1158/0008-5472.CAN-10-4660 PMC4295768

[mol212735-bib-0024] Lin Q , Lai R , Chirieac LR , Li C , Thomazy VA , Grammatikakis I , Rassidakis GZ , Zhang W , Fujio Y , Kunisada K *et al* (2005) Constitutive activation of JAK3/STAT3 in colon carcinoma tumors and cell lines: inhibition of JAK3/STAT3 signaling induces apoptosis and cell cycle arrest of colon carcinoma cells. Am J Pathol 167, 969–980. 16192633 10.1016/S0002-9440(10)61187-X PMC1603671

[mol212735-bib-0025] Lv D , Guo L , Zhang T and Huang L (2017) PRAS40 signaling in tumor. Oncotarget 8, 69076–69085. 28978182 10.18632/oncotarget.17299 PMC5620322

[mol212735-bib-0026] Masri J , Bernath A , Martin J , Jo OD , Vartanian R , Funk A and Gera J (2007) mTORC2 activity is elevated in gliomas and promotes growth and cell motility via overexpression of rictor. Cancer Res 67, 11712–11720. 18089801 10.1158/0008-5472.CAN-07-2223

[mol212735-bib-0027] Musa J , Orth MF , Dallmayer M , Baldauf M , Pardo C , Rotblat B , Kirchner T , Leprivier G and Grunewald TG (2016) Eukaryotic initiation factor 4E‐binding protein 1 (4E‐BP1): a master regulator of mRNA translation involved in tumorigenesis. Oncogene 35, 4675–4688. 26829052 10.1038/onc.2015.515

[mol212735-bib-0028] Ni Z , Lou W , Leman ES and Gao AC (2000) Inhibition of constitutively activated Stat3 signaling pathway suppresses growth of prostate cancer cells. Cancer Res 60, 1225–1228. 10728680

[mol212735-bib-0029] Panda AC , Martindale JL and Gorospe M (2017) Polysome fractionation to analyze mRNA distribution profiles. Bio Protoc 7, e2126. 10.21769/BioProtoc.2126 PMC5431591 28516123

[mol212735-bib-0030] Peterson TR , Laplante M , Thoreen CC , Sancak Y , Kang SA , Kuehl WM , Gray NS and Sabatini DM (2009) DEPTOR is an mTOR inhibitor frequently overexpressed in multiple myeloma cells and required for their survival. Cell 137, 873–886. 19446321 10.1016/j.cell.2009.03.046 PMC2758791

[mol212735-bib-0031] Raught B , Peiretti F , Gingras AC , Livingstone M , Shahbazian D , Mayeur GL , Polakiewicz RD , Sonenberg N and Hershey JW (2004) Phosphorylation of eucaryotic translation initiation factor 4B Ser422 is modulated by S6 kinases. EMBO J 23, 1761–1769. 15071500 10.1038/sj.emboj.7600193 PMC394247

[mol212735-bib-0032] Richter JD and Sonenberg N (2005) Regulation of cap‐dependent translation by eIF4E inhibitory proteins. Nature 433, 477–480. 15690031 10.1038/nature03205

[mol212735-bib-0033] Rong L , Livingstone M , Sukarieh R , Petroulakis E , Gingras AC , Crosby K , Smith B , Polakiewicz RD , Pelletier J , Ferraiuolo MA *et al* (2008) Control of eIF4E cellular localization by eIF4E‐binding proteins, 4E‐BPs. RNA 14, 1318–1327. 18515545 10.1261/rna.950608 PMC2441981

[mol212735-bib-0034] Samuels Y , Diaz LA Jr , Schmidt‐Kittler O , Cummins JM , Delong L , Cheong I , Rago C , Huso DL , Lengauer C , Kinzler KW *et al* (2005) Mutant PIK3CA promotes cell growth and invasion of human cancer cells. Cancer Cell 7, 561–573. 15950905 10.1016/j.ccr.2005.05.014

[mol212735-bib-0035] Saxton RA and Sabatini DM (2017) mTOR signaling in growth, metabolism, and disease. Cell 169, 361–371. 10.1016/j.cell.2017.03.035 28388417

[mol212735-bib-0036] Seeboeck R , Sarne V and Haybaeck J (2019) Current coverage of the mTOR pathway by next‐generation sequencing oncology panels. Int J Mol Sci 20, 690. 10.3390/ijms20030690 PMC6387057 30764584

[mol212735-bib-0037] Shahbazian D , Roux PP , Mieulet V , Cohen MS , Raught B , Taunton J , Hershey JW , Blenis J , Pende M and Sonenberg N (2006) The mTOR/PI3K and MAPK pathways converge on eIF4B to control its phosphorylation and activity. EMBO J 25, 2781–2791. 16763566 10.1038/sj.emboj.7601166 PMC1500846

[mol212735-bib-0038] She QB , Halilovic E , Ye Q , Zhen W , Shirasawa S , Sasazuki T , Solit DB and Rosen N (2010) 4E‐BP1 is a key effector of the oncogenic activation of the AKT and ERK signaling pathways that integrates their function in tumors. Cancer Cell 18, 39–51. 20609351 10.1016/j.ccr.2010.05.023 PMC3286650

[mol212735-bib-0039] Silvera D , Formenti SC and Schneider RJ (2010) Translational control in cancer. Nat Rev Cancer 10, 254–266. 20332778 10.1038/nrc2824

[mol212735-bib-0040] Sonenberg N and Hinnebusch AG (2009) Regulation of translation initiation in eukaryotes: mechanisms and biological targets. Cell 136, 731–745. 19239892 10.1016/j.cell.2009.01.042 PMC3610329

[mol212735-bib-0041] Sonenberg N , Morgan MA , Merrick WC and Shatkin AJ (1978) A polypeptide in eukaryotic initiation factors that crosslinks specifically to the 5'‐terminal cap in mRNA. Proc Natl Acad Sci USA 75, 4843–4847. 217002 10.1073/pnas.75.10.4843 PMC336217

[mol212735-bib-0042] Srivastava J and DiGiovanni J (2016) Non‐canonical Stat3 signaling in cancer. Mol Carcinog 55, 1889–1898. 26649644 10.1002/mc.22438

[mol212735-bib-0043] Thoreen CC , Chantranupong L , Keys HR , Wang T , Gray NS and Sabatini DM (2012) A unifying model for mTORC1‐mediated regulation of mRNA translation. Nature 485, 109–113. 22552098 10.1038/nature11083 PMC3347774

[mol212735-bib-0044] Truitt ML and Ruggero D (2016) New frontiers in translational control of the cancer genome. Nat Rev Cancer 16, 288–304. 27112207 10.1038/nrc.2016.27 PMC5491099

[mol212735-bib-0045] Xu J , Li X , Yang H , Chang R , Kong C and Yang L (2013) SIN1 promotes invasion and metastasis of hepatocellular carcinoma by facilitating epithelial‐mesenchymal transition. Cancer 119, 2247–2257. 23564492 10.1002/cncr.28023

[mol212735-bib-0046] Yokogami K , Wakisaka S , Avruch J and Reeves SA (2000) Serine phosphorylation and maximal activation of STAT3 during CNTF signaling is mediated by the rapamycin target mTOR. Curr Biol 10, 47–50. 10660304 10.1016/s0960-9822(99)00268-7

[mol212735-bib-0047] Yu H , Lee H , Herrmann A , Buettner R and Jove R (2014) Revisiting STAT3 signalling in cancer: new and unexpected biological functions. Nat Rev Cancer 14, 736–746. 25342631 10.1038/nrc3818

[mol212735-bib-0048] Zhang L , Gao L , Li Y , Lin G , Shao Y , Ji K , Yu H , Hu J , Kalvakolanu DV , Kopecko DJ *et al* (2008) Effects of plasmid‐based Stat3‐specific short hairpin RNA and GRIM‐19 on PC‐3M tumor cell growth. Clin Cancer Res 14, 559–568. 18223232 10.1158/1078-0432.CCR-07-1176

[mol212735-bib-0049] Zhang YJ , Dai Q , Sun DF , Xiong H , Tian XQ , Gao FH , Xu MH , Chen GQ , Han ZG and Fang JY (2009) mTOR signaling pathway is a target for the treatment of colorectal cancer. Ann Surg Oncol 16, 2617–2628. 19517193 10.1245/s10434-009-0555-9

[mol212735-bib-0050] Zhang Y and Zheng XF (2012) mTOR‐independent 4E‐BP1 phosphorylation is associated with cancer resistance to mTOR kinase inhibitors. Cell Cycle 11, 594–603. 22262166 10.4161/cc.11.3.19096 PMC3315097

