# Supplementary material for: STAT3‐mediated MLST8 gene expression regulates cap‐dependent translation in cancer cells
Source: Mol Oncol. 2020 Jun 29;14(8):1850–67. doi: 10.1002/1878-0261.12735 (PMC7400782; doi:10.1002/1878-0261.12735)
Supplement: Supplementary file 1 — Fig. S1. Time‐course changes of cell viability and protein level after STAT3 knockdown. Fig. S2. Effect of eIF4B knockdown on cap‐dependent translation. Fig. S3. The mRNA expression of mTORC1 components in STAT3 knockdown cells. Fig. S4. Effect of STAT3 knockdown on mTOR signaling in MLST8 knockdown or MLST8‐overexpressed HCT116 cells. Fig. S5. Effect of IL‐6 treatment on MLST8 gene expression in HCT116 cells. Fig. S6. Effect of STAT1 knockdown on MLST8 gene expression in HCT116 cells. Table S1. The siRNA target sequences. Table S2. The primer sequences for real‐time PCR. [file MOL2-14-1850-s001.docx]

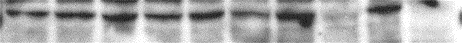

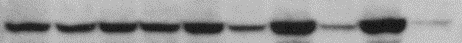

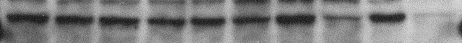

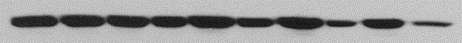

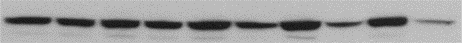


p-STAT3(Y705)

p-STAT3(S727)

GAPDH

HCT116

MDA-MB-231

siSTAT3:

Time:

–

+

–

+

–

+

–

+

–

+

6 h

12 h

24 h

48 h

72 h

–

+

–

+

–

+

–

+

–

+

6 h

12 h

24 h

48 h

72 h

**A**

**B**

STAT3


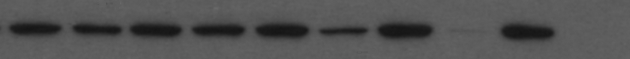


STAT3/GAPDH

1.0

0.8

1.0

0.9

1.0

0.5

1.1

0.1

1.0

0.0


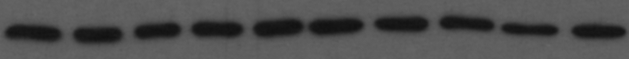


Y705/GAPDH

1.0

0.9

1.4

1.0

1.2

0.4

1.9

0.3

2.1

0.1

S727/GAPDH

1.0

0.9

1.2

0.9

1.0

0.7

1.2

0.3

1.1

0.0

1.0

0.9

0.8

0.8

1.0

0.9

1.4

0.5

1.0

0.2

1.0

1.0

1.3

1.2

1.6

1.2

2.1

0.5

1.6

0.1

1.0

0.6

0.8

0.8

0.9

0.9

1.3

0.5

0.8

0.1


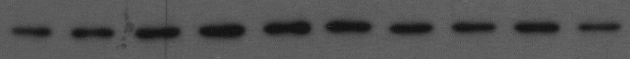

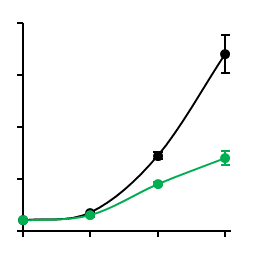

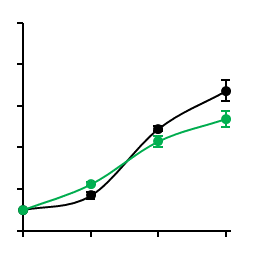


HCT116

MDA-MB-231

*

**

**

Relative Cell Proliferation

Relative Cell Proliferation

siRNA treatment (h)

siRNA treatment (h)

0

24

48

72

0

0.5

1.0

1.5

2.0

siSTAT3

siCTRL

1.0

0

0.6

0.8

0.4

0.2

0

24

48

72

*

**Fig. S1. Time-course changes of cell viability and protein level after STAT3 knockdown.** (A) Cell proliferation was measured with WST-1 assay daily after siRNA treatment (5 nM) in HCT116 and MDA-MB-231 cells (*n* = 3). (B) Protein extracts prepared at different time points after treatment with siCTRL (–) or siSTAT3 (+) were analyzed with Western blotting using indicated antibodies. The numbers under each blot indicate the relative amount of protein compared to the group treated with siCTRL for 6 h. Data are presented as mean ± SEM. Statistically significant differences are marked with *, *p* < 0.05; **, *p* < 0.01 (*t*-test).


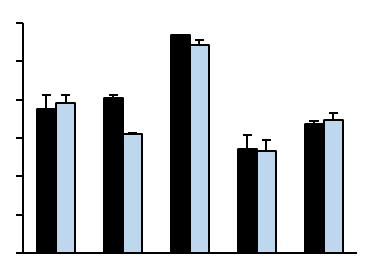


**A**

eIF4B

p-eIF4B(S422)

GAPDH


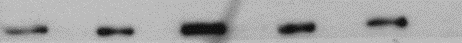

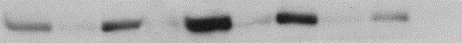


A549

ACHN

HCT116

LNCaP

MDA-

MB-231

siEIF4B:

Cells:

–

+

–

+

–

+

–

+

–

+


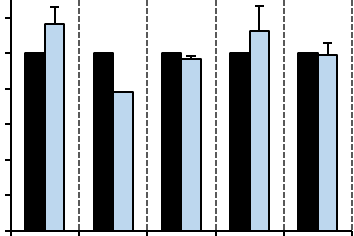

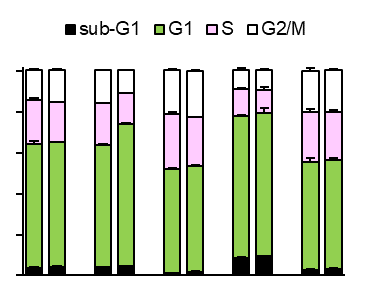

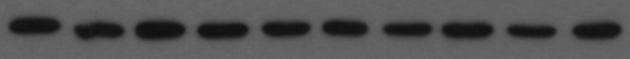


Cap-dependent

Translation (%)

***

0

60

40

80

20

100

120

**B**

A549

ACHN

HCT116

LNCaP

MDA-

MB-231

siCTRL

siEIF4B

**C**

0

60

40

80

20

100

**

A549

ACHN

HCT116

LNCaP

MDA-

MB-231

siCTRL

siEIF4B

120

Cell Number

( x 10^4^ cells)

**D**

***

A549

ACHN

HCT116

LNCaP

MDA-

MB-231

siEIF4B:

Cells:

–

+

–

+

–

+

–

+

–

+

0

60

40

80

20

100

Cell Cycle (%)


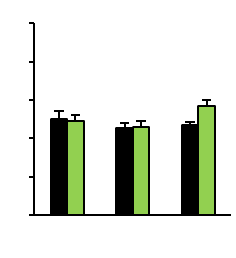


EIF4B mRNA level

0

1.2

0.8

1.6

0.4

2.0


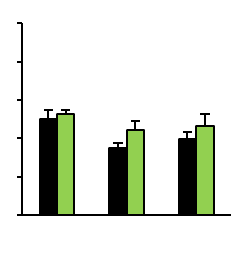

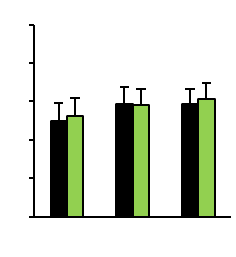


HCT116

ns

ns

*

siCTRL

siSTAT3

MDA-MB-231

ns

ns

ns

siCTRL

siSTAT3

EIF4B mRNA level

0

1.2

0.8

1.6

0.4

2.0

siRNA treatment (h)

siRNA treatment (h)

24

48

72

24

48

72

**E**

ACHN

ns

ns

ns

siCTRL

siSTAT3

siRNA treatment (h)

EIF4B mRNA level

0

1.2

0.8

1.6

0.4

2.0

24

48

72

**

**Fig. S2. Effect of eIF4B knockdown on cap-dependent translation.** (A) Western blotting was performed 72 h after siEIF4B treatment using indicated antibodies. (B) Cells were transfected with siRNA for 72 h. The cells were then transfected with a bicistronic luciferase reporter. The percent of cap-dependent translation was determined (*n* = 2). (C) Cell proliferation (*n* = 2) and (D) Distribution of cell cycle (*n* = 2) were measured 72 h after siEIF4B treatment. The siEIF4B group was statistically compared to the siCTRL group for each cell line. (E) The relative amounts of *EIF4B* mRNA were analyzed by qRT-PCR analysis using RNA from cells harvested at indicated time points. The siSTAT3 group was statistically compared to the siCTRL group at each point (*n*=4). Data are presented as mean ± SEM. Statistically significant difference is marked with *, *p* < 0.05; **, *p* < 0.01; ***, *p* < 0.001; ns, statistically insignificant (*t*-test).


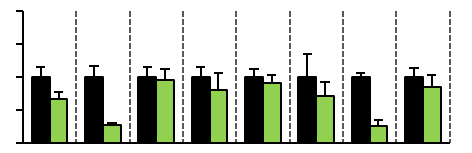

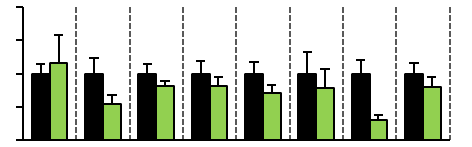

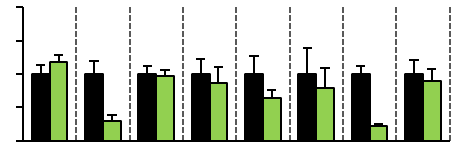

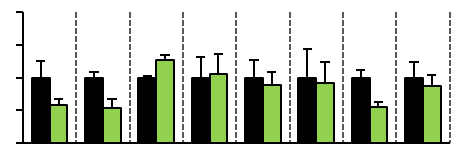

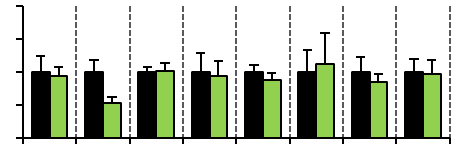


*

A549

***

ACHN

**

HCT116

LNCaP

TTI1

TELO2

RAPTOR

RAC1

PRAS40

PDIA3

MLST8

DEPTOR

*

*

***

*

*

*

*

siCTRL

siSTAT3

0

1.5

1.0

0.5

Ratio

2.0

0

1.5

1.0

0.5

Ratio

2.0

0

1.5

1.0

0.5

Ratio

2.0

0

1.5

1.0

0.5

Ratio

2.0

0

1.5

1.0

0.5

Ratio

2.0

MDA-MB-231

**Fig. S3. The mRNA expression of mTORC1 components in STAT3 knockdown cells.** The relative amounts of mRNA of mTORC1 components were analyzed by qRT-PCR analysis using RNA from cells harvested at 72 h after siRNA treatment. The siSTAT3 group was statistically compared to the siCTRL group at each point. Data are presented as mean ± SEM. Only statistically significant differences are marked with *, *p* < 0.05; **, *p* < 0.01; ***, *p* < 0.001 (*n* = 4) (*t*-test).

p-mTOR(S2448)

p-mTOR(S2481)

p-p70S6K(T389)

mTOR

p70S6K

AKT

p-AKT(T308)

p-AKT(S473)


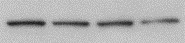

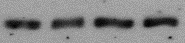

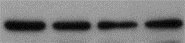

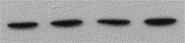

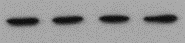

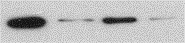

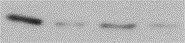

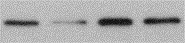

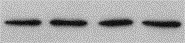

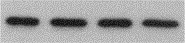

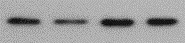

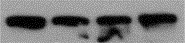

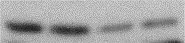

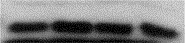

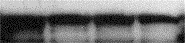


MLST8

HCT116

–

+

–

+

siSTAT3:

Cells:

HCT116

**A**

**B**

siSTAT3:

siMLST8:

–

+

–

+

–

+

–

+

p-mTOR(S2448)

p-mTOR(S2481)

p-p70S6K(T389)

mTOR

p70S6K

AKT

p-AKT(T308)

p-AKT(S473)

GAPDH


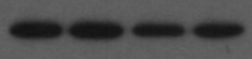


GAPDH


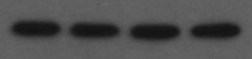

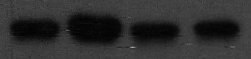


**Fig. S4. Effect of STAT3 knockdown on mTOR signaling in *MLST8* knockdown or MLST8-overexpressed HCT116 cells.** (A) siSTAT3 (5 nM) were transfected into cells for 48 h. The cells were then secondly transfected with siMLST8 (1 nM) for 24 h. Western blotting was performed using equal amounts of extracts with indicated antibodies. (B) siSTAT3 (2 nM) were transfected into HCT116 or MLST8-HCT116 cells for 72 h. Western blotting was performed using equal amounts of extracts with indicated antibodies.

IL-6:

STAT3

p-STAT3(S727)

p-STAT3(Y705)

mLST8

GAPDH

24

Time (h) :


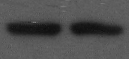

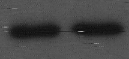

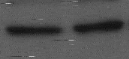

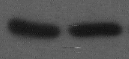

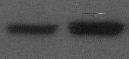


**A**


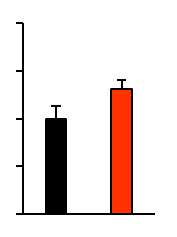

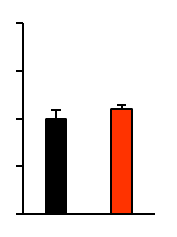


–

+

0.5

1.0

1.5

2.0

IL-6

*

CYP1B1 mRNA level

MLST8 mRNA level

0.0

0.5

1.0

1.5

2.0

IL-6

ns

**B**

–

+

–

+

**Fig. S5. Effect of IL-6 treatment on *MLST8* gene expression in HCT116 cells.** Cells were treated with IL-6 (10 ng/ml) for 24 h. (A) Protein extracts were prepared and Western blotting was performed using equal amounts of extracts with indicated antibodies. (B) The relative amounts of *MLST8* or *CYP1B1* mRNA were analyzed by qRT-PCR analysis using RNA from cells harvested. Data are presented as mean ± SEM. Statistically significant difference is marked with *, *p* < 0.05 (*n* = 5); ns, statistically insignificant (*t*-test).

**B**

24

siSTAT1:

Time (h) :

STAT1

mLST8

GAPDH

p-STAT1(Y701)

STAT3

p-STAT3(S727)

p-STAT3(Y705)


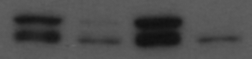

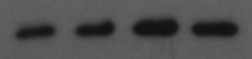

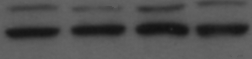

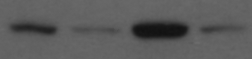

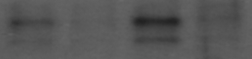

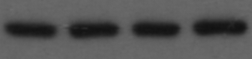

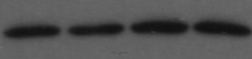


–

+

–

+

48

**A**


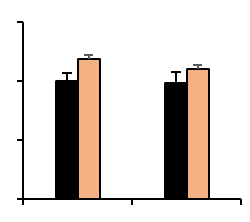


24

48

ns

ns

MLST8 mRNA level

0.0

0.5

1.0

1.5

siRNA treatment (h)

siCTRL

siSTAT1

**Fig. S6. Effect of STAT1 knockdown on *MLST8* gene expression in HCT116 cells.** Cells were treated with siSTAT1 (10 nM) for 24 h or 48 h. (A) Protein extracts were prepared and Western blotting was performed using equal amounts of extracts with indicated antibodies. (B) The relative amounts of *MLST8* mRNA were analyzed by qRT-PCR analysis using RNA from cells harvested at indicated time points. Data are presented as mean ± SEM. The siSTAT1 group was statistically compared to the siCTRL group at each point (*n* = 2); ns, statistically insignificant (*t*-test).

**Table S1**. The siRNA target sequences

| Genes | siRNA Name | Sequence (5’ to 3’) |
| --- | --- | --- |
| *EIF4EBP1* | si4EBP1 | UCGGAACUCACCUGUGACCAA |
| *EIF4B* | siEIF4B | GGACAGGAAGUGAGUCAUC |
| *MLST8* | siMLST8 | AGCGGAUCUUCCAGGUGAA |
| *STAT1** | siSTAT1-1 | AACUAGUGGAGUGGAAGCGGA |
|  | siSTAT1-2 | AAGGAAGUAGUUCACAAAAUA |
| *STAT3** | siSTAT3-1 | AACAUCUGCCUAGAUCGGCUA |
|  | siSTAT3-2 | CAGCCUCUCUGCAGAAUUCAA |

*Equimolar siSTAT1-1 and siSTAT1-2 were combined for *STAT1* and siSTAT3-1 and siSTAT3-2 were combined for *STAT3* knockdown, respectively.

**Table S2**. The primer sequences for real-time PCR

| Genes | Sense (5’ to 3’) | Antisense (5’ to 3’) |
| --- | --- | --- |
| *DEPTOR* | catcatccagcatgtgtcca | ttgtcatggctctgcttcct |
| *CCND1* | AATCCGCCCTCCATGGTG | CCAGCAGGGCTTCGATCTG |
| *CCND3* | AGACCTTTTTGGCCCTCTGT | AGCTTCGATCTGCTCCTGAC |
| *GAPDH* | ggaaggactcatgaccacagt | cagtgagcttcccgttcag |
| *MLST8* | ctgtcaatagcaccggaaac | gtctgatcagccgagcaggt |
| *PDIA3* | aacttggcgagaagctcagc | attcacggccaccttcatat |
| *POLR2A* | GCACCACGTCCAATGACAT | GTGCGGCTGCTTCCATAA |
| *PRAS40* | agtgataatggagggctctt | acttggcgtactgctgtgtg |
| *RAC1* | atgtccgtgcaaagtggt | accctgcggataggtgat |
| *RAPTOR* | atggagtccgaaatgctg | actgactgtcttcatccgat |
| *TELO2* | gacagccgggatgaactgct | cagcagctcgaggctcagtt |
| *TTI1* | aaatccgcctgaaggtcttg | ttgaaggctctaagcactgc |
